# From principles to binding rules: A one health governance readiness analysis of Saudi Arabia's AI health policy against global regulatory benchmarks

**DOI:** 10.1016/j.onehlt.2026.101570

**Published:** 2026-09-12

**Authors:** Ahmed Alqheedan

**Affiliations:** Department of Health Informatics, College of Health Sciences, Saudi Electronic University, Riyadh, Saudi Arabia

**Keywords:** Artificial intelligence governance, Public health policy, Saudi Arabia, Regulatory gap analysis, EU AI Act, Health data interoperability

## Abstract

**Background:**

AI is entering clinical care, disease surveillance, and health-data systems faster than governance frameworks can accommodate. Stakes for public health systems include algorithmic safety in diagnosis and triage, data interoperability, and workforce capacity to supervise AI tools safely. Saudi Arabia has built substantial AI health governance infrastructure through its Data and Artificial Intelligence Authority and Food and Drug Authority, but no binding, AI-specific health law had been enacted as of mid-2026.

**Methods:**

A ten-dimension governance scoring framework anchored in WHO and EU AI Act standards covered legal bindingness, risk classification, health-specific AI provisions, transparency, post-market surveillance, data governance/interoperability, independent auditing, codified ethics, and workforce/capacity building. Nineteen policy documents across Saudi Arabia, the EU, the USA, and China were scored 0–2 (absent, partial/non-binding, codified/binding), citing source passages. A genuine second coder was unavailable; reliability was assessed via single-coder self-consistency on a random subsample, and a separate post-hoc construct-validity review checked cross-jurisdictional consistency of rubric application. RTGI (Regulatory Transition Gap Index) was computed under two weighting schemes and stress-tested with a randomized-weight sensitivity sweep.

**Results:**

Saudi Arabia scored 15 of 20 (RTGI = 0.25), second only to the EU (16/20, RTGI = 0.20) and ahead of the USA (14/20, RTGI = 0.30) and China (6/20, RTGI = 0.70; sensitivity band 5–11/20 accounting for translation limitations). Rank order was stable across weighting schemes and a 1000-draw randomized-weight sensitivity sweep (China's largest-gap position held in 99.3% of draws; full baseline order reproduced in 53.5% of draws). Single-coder self-consistency agreement on a random subsample was 83.3% (kappa = 0.64, substantial). Saudi Arabia's largest gaps were independent third-party conformity assessment, data governance/interoperability, codified ethics, and workforce/capacity building.

**Conclusions:**

Saudi Arabia's AI health governance is strong on device-specific technical guidance but relies on internal quality-management auditing rather than independent third-party conformity assessment, and has not converted ethics principles into binding rules with the public-health reach of the EU AI Act. Four near-term actions are identified to close the highest-priority gaps.

## 1 Introduction

AI is entering clinical practice, generating diagnostic and treatment recommendations, faster than health systems have built regulatory frameworks to govern it. Clinical AI raises governance questions beyond generic AI regulation: safety and post-market surveillance, human oversight of algorithmic decisions, and interoperability of AI-generated data across care settings. Unregulated deployment can propagate errors across populations, fragment the health data infrastructure disease surveillance depends on, and outpace workforce capacity to supervise algorithmic outputs.

Saudi Arabia has assembled substantial AI governance architecture over four years. SDAIA published national AI Ethics Principles in 2023 [1] and generative-AI guidelines in 2024 [2], [3]. The SFDA issued MDS-G010, guidance on AI/ML-based medical devices, in 2023 [4], followed by broader medical device software guidance (MDS-G027) in 2025 [5]. The PDPL, in force since 2023, applies to health data processed by AI systems [6]. A draft Responsible AI Policy entered public consultation in early 2026 [7].

Prior comparative literature finds SFDA guidance draws on established international frameworks rather than independent paradigms [8], proposes a “true lifecycle approach” using the GCC as a governance model [9], [10], and finds GCC national AI strategies emphasize soft, principles-based instruments over binding rules, risking ethics washing where principles lack enforcement [11], [12]. Further work examines PDPL alongside government AI adoption [13], clinical AI liability across MENA [14], and PDPL against GDPR, the EU AI Act, and the European Health Data Space Regulation, concluding PDPL is formally more protective than GDPR but structurally less enabling of data-intensive clinical AI [15]. No prior work applies a structured, multi-dimensional gap-scoring framework benchmarked against WHO guidance and the EU AI Act to Saudi Arabia's full AI health policy ecosystem since August 2024.

These governance gaps are not confined to individual clinical encounters. The same interoperability and transparency provisions that govern AI-assisted diagnosis also underpin AI-enabled disease surveillance, antimicrobial-resistance monitoring, and population-level environmental health risk assessment, the cross-sectoral surveillance capacity a One Health approach treats as interdependent with clinical care. A governance framework that leaves health-data interoperability partially codified, as this analysis finds for Saudi Arabia, constrains not only clinical AI oversight but the disease-surveillance infrastructure that depends on the same data-sharing and transparency mechanisms.

The OECD reported in 2026 that only 18% of OECD member countries had adopted a national AI-in-healthcare strategy, with 32% working toward one [16]. Saudi Arabia's position relative to this benchmark, and to jurisdictions with more codified AI health law, is the empirical question this study addresses. This analysis involves no human subjects and requires no institutional review board approval; all data are publicly available regulatory and policy texts.

## 2 Research objectives

Four objectives structure the analysis: (O1) construct a normative, ten-dimension AI health governance scoring framework grounded in WHO and EU AI Act standards; (O2) apply the framework to Saudi Arabia's AI health policy ecosystem; (O3) benchmark Saudi Arabia against three comparators, the EU, the United States, and China; (O4) rank highest-priority governance gaps into a policy roadmap aligned with Vision 2030 health transformation objectives.

## 3 Theoretical framework

This analysis draws on Baldwin and Cave's regulatory ladder [17], which treats governance instruments as moving from voluntary principles, through non-binding guidance, to enforceable rules with independent verification. A jurisdiction can occupy different rungs simultaneously: it may codify ethical principles while leaving post-market surveillance voluntary. The scoring framework operationalizes this ladder by coding each dimension as absent (0), partial/non-binding (1), or codified/binding (2).

Two normative anchors ground the scoring dimensions. WHO guidance on ethics and governance of large multi-modal models for health [18] identifies transparency, accountability, and human oversight as central requirements. The EU AI Act [19] operationalizes comparable principles into a risk-tiered, binding structure, classifying medical-device AI as high-risk with transparency, monitoring, and conformity-assessment obligations. One anchor sets the ethical baseline; the other provides the enforcement-grade benchmark.

## 4 Methods

### 4.1 Study design

Original comparative policy document analysis using a deductive approach: a scoring framework defined a priori from normative sources was applied to primary policy texts. No human subjects or institutional review board approval required.

### 4.2 Policy document corpus

Nineteen documents across four jurisdictions plus two normative anchors (Table 1) were identified from official regulator domains or the Internet Archive's Wayback Machine. Two Saudi documents were retrievable only as secondary reporting; scores were capped at 1. Supplementary Table S2 records, for each document, an official document date rather than a crawler-access timestamp: for versioned regulatory documents this is the date stamped on the document itself (e.g., SFDA guidance carries an internal version code encoding its issue date), and otherwise the officially reported publication or effective date, each with its supporting source cited in the table. Chinese-language NHC guidance and the NMPA notice were coded from non-professional translation. Reflecting this, none of China's ten scored cells carried high confidence (five medium, five low; Supplementary Table S1). A sensitivity band applying a ±1-point perturbation (bounded 0–2) to the five low-confidence cells, holding the five medium-confidence cells fixed, yields a raw-score range of 5–11 (of 20) against the primary-coding value of 6; RTGI ranges 0.45–0.75 (equal-weighted). China's score remains below the USA's (14/20) across the full band, so China's ranking as the largest regulatory transition gap is not sensitive to this translation limitation.

**Table 1:** Policy document inventory.

Jurisdiction	Document	Year	Binding status	Retrieval status
Saudi Arabia	SFDA MDS-G010: AI/ML-Based Medical Device Guidance	2023	Regulatory guidance (compliance benchmark for market authorization)	Retrieved
Saudi Arabia	SFDA MDS-G027: Medical Device Software Guidance	2025	Regulatory guidance (compliance benchmark)	Retrieved
Saudi Arabia	SDAIA AI Ethics Principles v1.0	2023	Non-binding (voluntary ethics framework)	Retrieved
Saudi Arabia	SDAIA Generative AI Guidelines for Government Entities	2024	Non-binding (institutional guidance)	Retrieved
Saudi Arabia	SDAIA Generative AI Guidelines for the Public	2024	Non-binding (public guidance)	Retrieved
Saudi Arabia	Draft Responsible AI Policy	2026	Not yet enacted (public consultation stage)	Secondary-only
Saudi Arabia	Personal Data Protection Law (PDPL) and Implementing Regulation	2023	Binding (national law)	Retrieved
Saudi Arabia	National Strategy for Data and AI (NSDAI)	2020	Non-binding (strategy document)	Secondary-only
Saudi Arabia	SDAIA AI Adoption Framework	2024	Non-binding (guidance)	Retrieved
European Union	Regulation (EU) 2024/1689 (AI Act)	2024	Binding (regulation, directly applicable)	Retrieved
European Union	MDCG 2025–6 guidance (MDR/IVDR-AI Act interplay)	2025	Binding-adjacent (official coordination-group guidance)	Retrieved
European Union	Regulation (EU) 2025/327 (European Health Data Space)	2025	Binding (regulation)	Retrieved
United States	FDA AI-Enabled Device Software Functions / PCCP guidance	2025	Non-binding (“nonbinding recommendations”, de facto regulatory expectation)	Retrieved (final supersedes cited 2023 draft)
United States	FDA Clinical Decision Support Software guidance	2026	Non-binding (“nonbinding recommendations”)	Retrieved (cover date differs from proposal citation)
United States	ONC HTI-1 Final Rule	2024	Binding (federal rule)	Retrieved
China	NMPA/CMDE Notice No. 8 (2022) on AI medical device registration review guideline	2022	Binding (regulator notice; attached guideline document itself untranslated)	Untranslated (partial)
China	NHC/NATCM/NDCPA Reference Guide for AI Application Scenarios in Health	2024	Non-binding (reference guide, 84 scenarios / 4 domains)	Untranslated
Normative anchor	WHO Guidance on Large Multi-Modal Models	2024	Normative (non-binding international guidance)	Retrieved
Normative anchor	OECD Scaling Artificial Intelligence in Health	2026	Normative (policy report, includes OECD member checklist)	Retrieved

### 4.3 Governance scoring framework

Ten governance dimensions from WHO and EU AI Act normative anchors were scored 0–2 per jurisdiction:

• **0 = Absent.** No identifiable provision addresses the dimension.
• **1 = Partial or non-binding.** Addressed by guidance, voluntary principles, strategy documents, or draft instruments, or addressed only in general terms where health-specific coverage was required.
• **2 = Codified or binding.** Addressed by enacted law, regulation, or compliance-benchmark regulatory guidance with health-specific scope.

Dimensions were: (1) legal/binding status of AI health rules [EU AI Act Article 6]; (2) risk classification for health AI [EU AI Act Annex III; WHO]; (3) health-specific AI provisions beyond general coverage [WHO; OECD]; (4) generative AI in clinical settings [WHO]; (5) algorithmic transparency and explainability [EU AI Act Article 13]; (6) post-market surveillance and monitoring [EU AI Act Article 72; FDA]; (7) health data governance and interoperability [EU AI Act recitals; PDPL]; (8) independent auditing and third-party conformity assessment [EU AI Act Article 43]; (9) ethical principles, codified [WHO; SDAIA]; (10) workforce and capacity-building [OECD; WHO].

Secondary, draft, or consultation-stage sources were capped at score 1. Dimensions 2, 3, 4, and 6 required health-specific coverage; the rest could be satisfied by general cross-sectoral instruments.

### 4.4 Evidence extraction and scoring procedure

Primary text from 19 documents in 21 source files (15 PDFs, 6 HTML/XML) yielded over 3.4 million characters with page-level provenance. Keyword-anchored passage retrieval produced 30–48 candidate passages per jurisdiction (Saudi Arabia 43, EU 48, USA 42, China 30).

Each of 40 jurisdiction-by-dimension cells was scored independently against the rubric with source passages, page numbers, and stated confidence. Ten low-confidence cells underwent expanded second-pass keyword search; one score changed (United States, independent auditing, 0 to 1).

### 4.5 Reliability: self-consistency check and construct-validity review

A genuine second coder was unavailable for this solo-authored study. Reliability was assessed via single-coder test-retest: the original coder re-scored a random 30% subset (12 of 40 cells, fixed seed) blind to its own prior scores, with shuffled evidence passages. Agreement was 83.3% (10/12), Cohen's kappa = 0.64 (substantial, Landis and Koch 1977). Two disagreements were adjudicated: Saudi Arabia's health-data-governance dimension (retained at 1) and the United States' independent-auditing dimension (revised 1 to 0; ONC HTI-1 describes forward-looking policy, not an adopted requirement). This test-retest procedure measures within-coder stability over time; it does not substitute for independent inter-rater reliability, and this is treated as a limitation throughout (Section 7).

A separate post-hoc construct-validity check, distinct from the reliability procedure above, identified an inconsistency in dimension 8 (independent auditing and third-party conformity assessment). Saudi Arabia's original score of 2 was supported by SFDA MDS-G010 language on internal and external audits within quality-management-system (QMS) requirements, nonconformity evaluation, and CAPA verification. This is standard QMS-internal auditing and does not evidence the independent, external third-party conformity assessment the dimension is anchored to (EU AI Act Article 43; MDCG 2025–6). The same evidentiary standard applied to the United States on this dimension (self-consistency adjudication, above) treats forward-looking or non-adopted third-party mechanisms as insufficient; applying it consistently to Saudi Arabia revises the score from 2 to 1 (partial/non-binding: an auditing practice is evidenced, but not a binding requirement for independent third-party verification). This revision is carried through Table 2, Table 3, Table 4 and the RTGI calculations below.

**Table 2:** Governance dimension scores by jurisdiction (0 = absent, 1 = partial/non-binding, 2 = codified/binding).

Governance dimension	Saudi Arabia	EU	USA	China
Legal/binding status	2	2	1	1
Risk classification	2	2	2	0
Health-specific provisions	2	2	2	1
Generative AI in clinical use	1	0	1	1
Transparency & explainability	2	2	2	0
Post-market surveillance	2	2	2	1
Data governance & interoperability	1	1	2	1
Independent auditing	1	2	0	0
Ethical principles	1	1	2	0
Workforce & capacity building	1	2	0	1

**Table 3:** Regulatory Transition Gap Index (RTGI) by jurisdiction.

Jurisdiction	Raw score	Max possible	RTGI (equal-weighted)	RTGI (alternate-weighted)	Rank	Interpretation
China	6	20	0.70	0.7143	1	High regulatory transition gap (mostly absent/non-binding)
USA	14	20	0.30	0.3214	2	Moderate regulatory transition gap
Saudi Arabia	15	20	0.25	0.2143	3	Low-moderate regulatory transition gap
EU	16	20	0.20	0.1429	4	Low regulatory transition gap (mostly codified/binding)

**Table 4:** Saudi Arabia priority gaps, ranked.

Dim #	Governance dimension	Saudi score	Best comparator score	Best comparator(s)	Raw gap	Weighted gap	Priority rank
8	Independent auditing & third-party conformity	1	2	EU	1	2	1
7	Health data governance & interoperability	1	2	USA	1	1	2
9	Ethical principles (codified)	1	2	USA	1	1	2
10	Workforce & capacity building provisions	1	2	EU	1	1	2
1	Legal / binding status of AI health rules	2	2	EU	0	0	5
2	Risk classification system for health AI	2	2	EU, USA	0	0	5
3	Health-specific AI provisions (beyond general)	2	2	EU, USA	0	0	5
4	Generative AI in clinical settings	1	1	USA, China	0	0	5
5	Algorithmic transparency & explainability	2	2	EU, USA	0	0	5
6	Post-market surveillance & monitoring	2	2	EU, USA	0	0	5

Gap weighting follows the alternate scheme (Section 4.6), which double-weights legal/binding status, risk classification, post-market surveillance, and independent auditing. The weighted gap is (dimension weight) x (best comparator score - Saudi Arabia score).

### 4.6 Regulatory transition gap index (RTGI) and sensitivity analysis

RTGI was computed as the mean of (2 - score) / 2 across ten dimensions, a 0–1 scale (0 = no gap, 1 = complete gap). Equal-weighted and alternate-weighted RTGI (double-weighting legal/binding status, risk classification, post-market surveillance, and independent auditing) were compared via Spearman's rank correlation. RTGI was also recomputed across 1000 Dirichlet-random dimension-weight draws per jurisdiction, with each draw's rank order compared to the baseline (equal-weighted) ordering via Spearman correlation.

### 4.7 Priority gap ranking

Gap score per dimension was the difference between Saudi Arabia's score and the best comparator (EU, USA, China). Gaps were weighted using the alternate scheme from 4.6 and ranked to identify Saudi Arabia's highest-priority governance dimensions.

## 5 Results

### 5.1 Governance scoring matrix

Table 2 presents the ten-dimension, four-jurisdiction scoring matrix; complete evidentiary citations, rationale, and confidence ratings for all 40 cells appear in Supplementary Table S1.

Saudi Arabia scored 15/20, second only to the EU (16/20). Saudi Arabia scored 2 on legal/binding status, risk classification, health-specific provisions, transparency, and post-market surveillance; 1 on generative AI in clinical use, data governance, independent auditing, ethical principles, and workforce/capacity building. The independent-auditing score reflects binding internal quality-management auditing rather than independent third-party conformity assessment (Section 4.5). China scored 1 on generative AI in clinical use and health-specific provisions (NHC's 84-scenario guidance) but 0 on risk classification, transparency, independent auditing, and ethical principles.

### 5.2 Comparative governance profiles (Fig. 1)

**Fig. 1:**
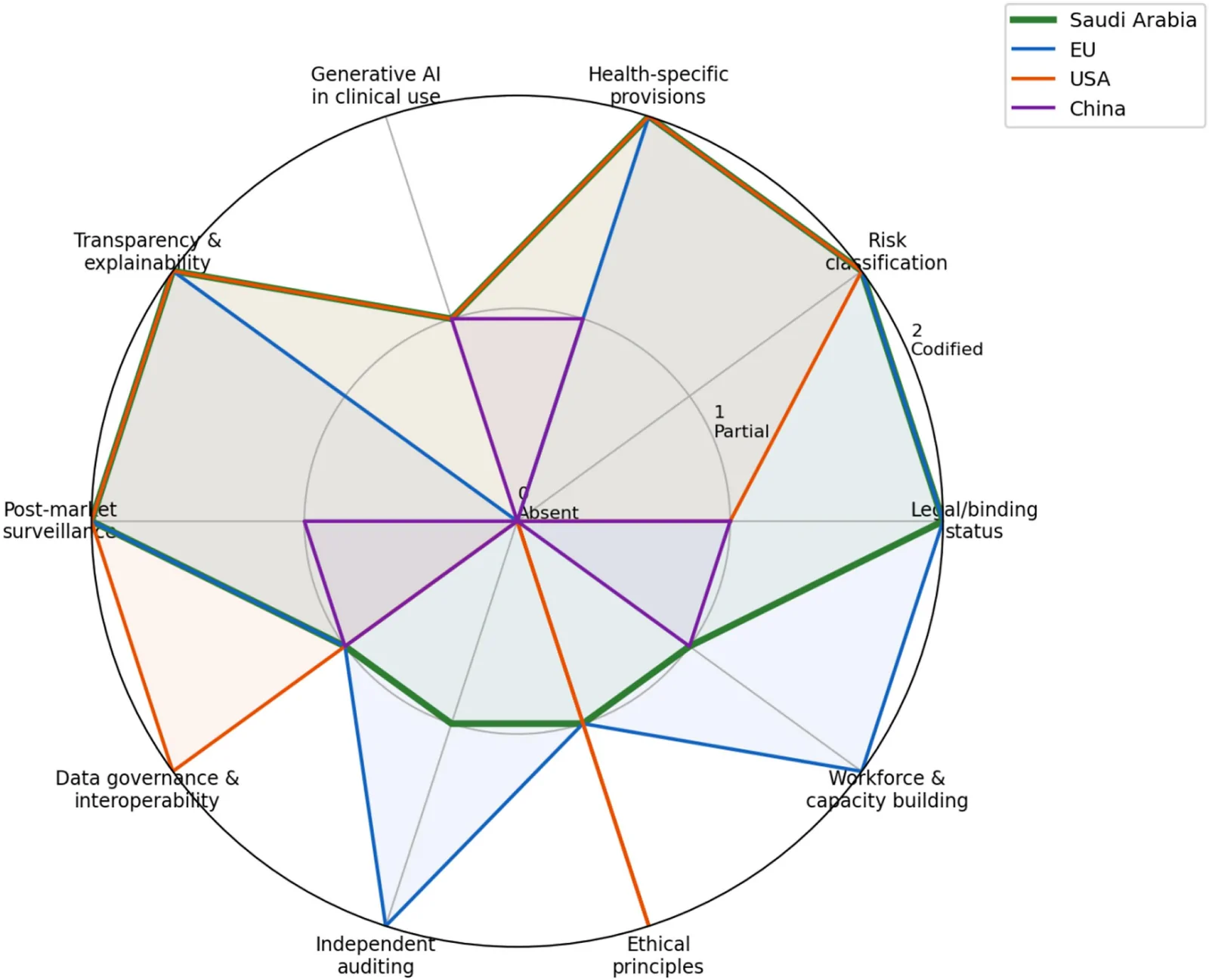
Governance profiles across ten dimensions: Saudi Arabia vs. EU, USA, and China.

Fig. 1 overlays all four profiles on a radar chart. Saudi Arabia and the EU overlap closely on legal status, risk classification, transparency, and post-market surveillance, but diverge on independent auditing, where Saudi Arabia's internal QMS-based provisions fall short of the EU's third-party conformity-assessment regime. The USA relies on non-binding guidance treated as de facto expectation. China's profile is narrowest, limited to scenario-coverage dimensions.

### 5.3 Regulatory transition gap index (Table 3)

China shows the largest gap under both schemes (RTGI 0.70 equal-weighted, 0.7143 alternate-weighted); the USA is intermediate (0.30/0.3214); Saudi Arabia (0.25/0.2143) trails the EU (0.20/0.1429) but leads the USA. Rank order is stable across weighting schemes and no longer produces a Saudi Arabia-EU tie under either scheme.

**Fig. 3:**
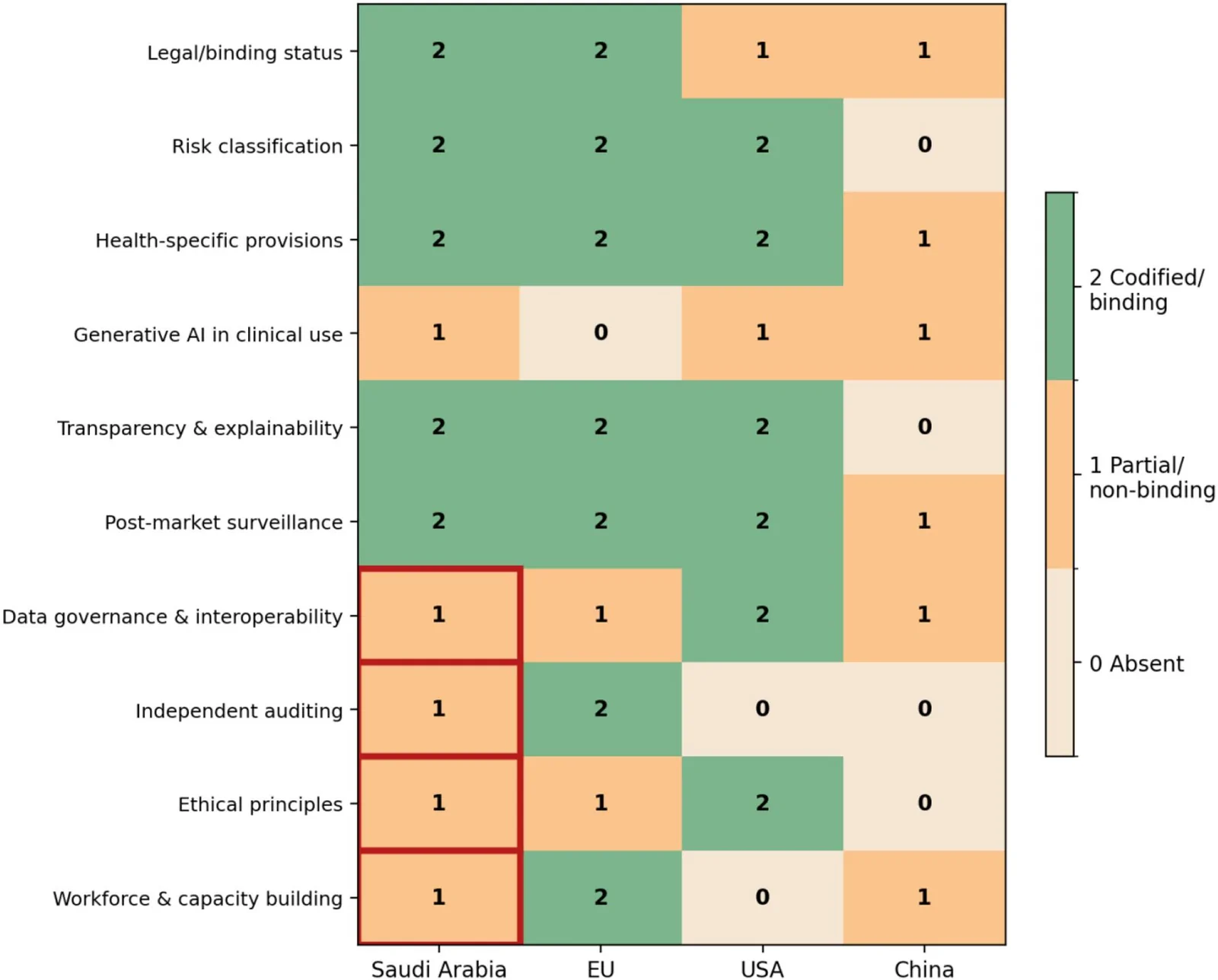
RTGI distributions across 1000 random dimension-weight draws, with baseline (equal-weighted) values marked.

A 1000-draw random dimension-weight sensitivity sweep (Fig. 3), re-executed against the corrected scoring matrix, tested stability beyond these two schemes. China's highest-gap position held in 99.3% of draws. With the Saudi Arabia-EU tie resolved, the full baseline order (China > USA > Saudi Arabia > EU) was reproduced exactly in 53.5% of draws, and the median Spearman rho against baseline was 1.000, both improvements on the pre-correction matrix, since a strict ranking is inherently more stable under weight perturbation than a tie.

Reliability rests on a single-coder self-consistency check (Section 4.5): re-scoring a 12-cell subsample blind to prior scores gave 83.3% agreement (kappa = 0.64, substantial). A genuine second coder was unavailable; this is disclosed as a limitation (Section 7).

### 5.4 Saudi Arabia's gap profile (Fig. 2)

**Fig. 2:**
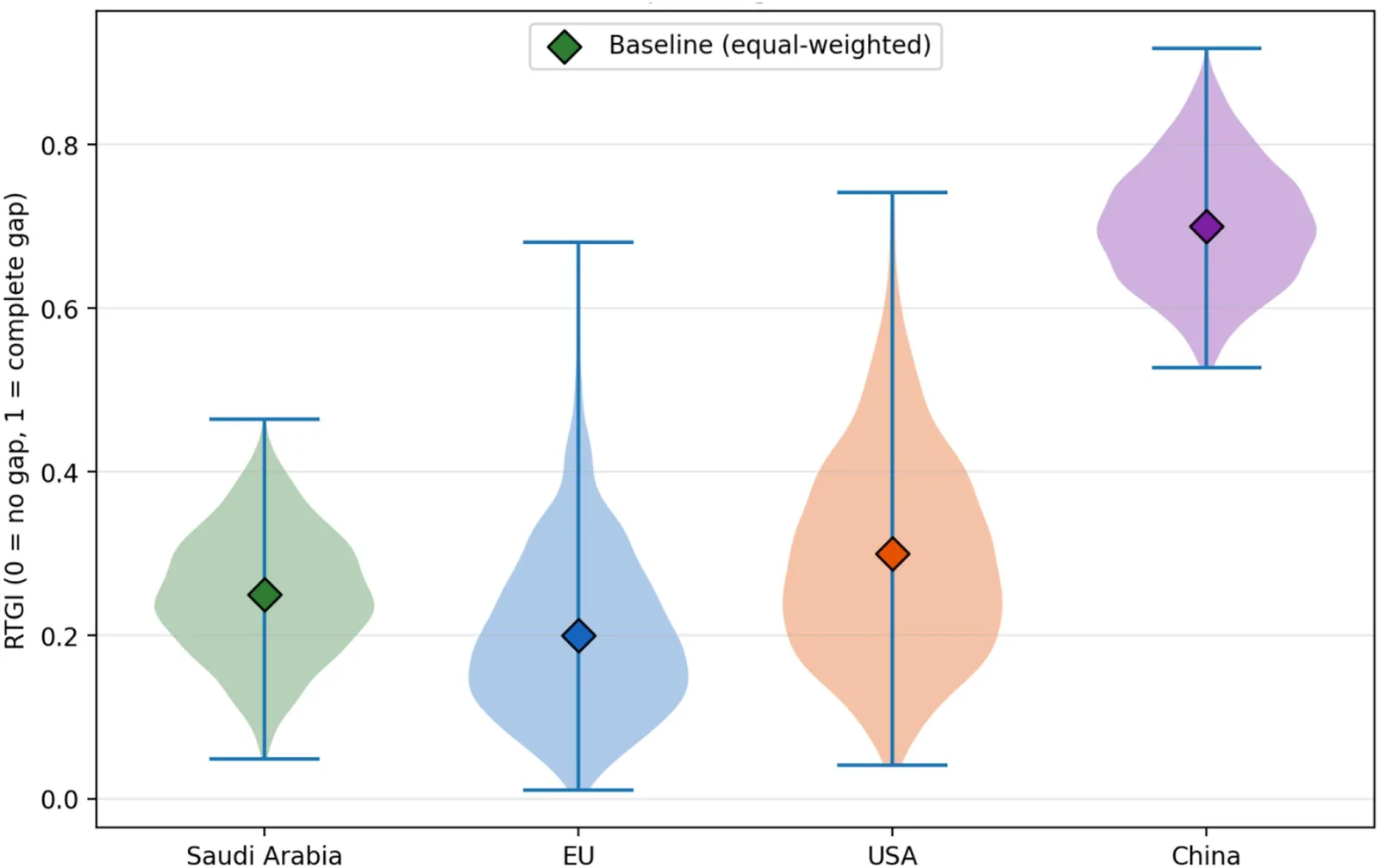
Saudi Arabia's governance gaps relative to global benchmarks. Outlined cells mark the top four priority gaps.

Fig. 2 plots Saudi Arabia's scores against the strongest comparator in each dimension, with the four largest gaps outlined.

### 5.5 Priority gap ranking (table 4)

Independent auditing carries the highest priority once correctly weighted: its double weighting in the alternate scheme means Saudi Arabia's shortfall there (internal QMS auditing rather than independent third-party conformity assessment) counts twice as heavily as the three tied second-priority gaps, data governance/interoperability via the USA's HTI-1 provisions, codified ethical principles via the USA's HTI-1 transparency requirements, and workforce/capacity building via the EU's AI Act recital language on AI-literacy obligations. The remaining six dimensions show no gap.

## 6 Discussion

### 6.1 Saudi Arabia's position on the regulatory ladder

Section 3's regulatory ladder predicts AI health governance instruments move from voluntary principles toward enforceable rules with independent verification as a jurisdiction matures. Saudi Arabia scores second only to the European Union on this ladder, ahead of the United States and China, qualifying claims that GCC states remain mainly at the principles stage [11], [12]. SFDA's MDS-G010 and MDS-G027 [4], [5] carry enforcement consequences through device marketing authorization, scoring as binding on legal status, risk classification, health-specific provisions, transparency, and post-market surveillance. The one dimension where Saudi Arabia's SFDA guidance falls short of the EU's binding floor is independent third-party conformity assessment: SFDA's audit provisions are internal, quality-management-system requirements rather than the external, notified-body verification the EU AI Act mandates (Article 43), a specific, addressable gap rather than a general shortfall in enforcement architecture. Adopting an international standard as domestic enforceable rule places a regulator on the upper rungs regardless of technical content origin [8].

### 6.2 The United States: guidance without a binding floor

The US scores lower than Saudi Arabia and the EU because its core instruments, FDA's Predetermined Change Control Plan and Clinical Decision Support guidance [20], [21], are formally non-binding under the Administrative Procedure Act, consistent with critiques that FDA regulation of AI/ML-based software was not built for continuously updating algorithms [22]. The US outperforms both on health data governance and interoperability through ONC's HTI-1 Final Rule [23], with no codified Saudi analogue at present.

### 6.3 China: descriptive scope without binding enforcement architecture

China's low RTGI-implied ranking reflects the reviewed corpus, not an absence of Chinese AI health activity. NHC's 84-scenario reference guide [24] catalogs prospective health AI applications but does not establish binding risk classification, independent audit, or codified ethical-principle requirements comparable to the other corpora. NMPA's 2022 notice on AI/ML medical device registration review [25] is binding, but only the notice text, not its attached technical guideline, was accessible during corpus construction (Section 4.2), which may understate China's true binding-rule coverage.

### 6.4 Interpreting Saudi Arabia's four priority gaps

The four dimensions in Table 4, independent third-party conformity assessment, health data governance and interoperability, codified ethical principles, and workforce/capacity building, share one feature: the strongest comparator converts a previously voluntary or internal commitment into an independently verifiable, binding requirement. Independent auditing carries the largest weighted gap: SFDA's audit provisions remain internal quality-management requirements, whereas the EU AI Act's Article 43 mandates external, notified-body conformity assessment (Section 6.1). For data governance, the US HTI-1 rule imposes binding transparency and interoperability obligations; the EU's European Health Data Space regulation [26] fills an interoperability-governance gap in EU health law [27]. Saudi Arabia's PDPL lacks a comparable codified requirement.

SDAIA's AI Ethics Principles remain voluntary, a structure common across national AI ethics documents and flagged as open to ethics washing [12], [28]. Convergence on shared principles has not produced binding accountability mechanisms [29], echoed in work on the gap between stated AI accountability principles and operationalization [30]. The US scores higher because transparency obligations sit within the codified HTI-1 rule.

For workforce provisions, the EU AI Act's recitals establish AI-literacy obligations for providers and deployers in health care [19]. No equivalent codified requirement exists in the Saudi corpus, despite policy attention to workforce development under Vision 2030 [31], [32], [33].

### 6.5 Policy implications

Four actions follow. First, establish an independent third-party conformity-assessment mechanism for AI-based medical devices, for example SFDA-accredited certification bodies analogous to the EU's notified-body system, since current SFDA guidance evidences binding internal quality-management auditing but not independent external verification, the dimension on which Saudi Arabia now shows its largest weighted gap. Second, extend the PDPL or add a health-data annex to the draft Responsible AI Policy with binding interoperability requirements. Third, convert select SDAIA AI Ethics Principles into binding requirements via SFDA marketing-authorization criteria, explicitly incorporating liability and patient-redress mechanisms for AI-attributable clinical harm, an accountability dimension absent from the ethical-principles instruments reviewed [29], [30]. Fourth, embed AI-literacy requirements into Vision 2030 health workforce programs. The remaining six dimensions show no measurable gap.

Because health-data interoperability and independent verification are also prerequisites for reliable cross-sectoral disease surveillance and antimicrobial-resistance monitoring, closing the auditing and data-governance gaps identified here carries value beyond clinical AI oversight: it strengthens the evidentiary and institutional foundation that One Health surveillance systems require to function across human, animal, and environmental health data streams [34].

## 7 Limitations

Reliability rests on a single-coder self-consistency check (Section 4.5): re-scoring a random 12-cell subsample blind to prior scores gave 83.3% agreement (kappa = 0.64, substantial). A genuine second coder was unavailable for this solo-authored study. Test-retest measures within-coder stability over time; it does not capture shared interpretive bias, cultural assumptions, or the influence of the coder's own prior beliefs about the jurisdictions scored, which only an independent second coder could reveal. The remaining 28 cells were not resampled and lack direct reliability evidence. This is the single most significant methodological limitation of the study, and the findings should be read with that constraint in mind. A separate post-hoc construct-validity review identified one dimension, independent auditing and third-party conformity assessment, where the original coding standard had been applied inconsistently across jurisdictions; Saudi Arabia's score was revised from 2 to 1 to align with the stricter standard already applied to the United States on the same dimension (Section 4.5). This revision was not detected by the self-consistency check, since it scored the original rubric application rather than its cross-jurisdictional consistency; it points to construct validity, not inter-rater or intra-rater reliability, as a distinct and necessary check alongside them, and underscores that a single coder, however careful, cannot substitute for independent replication.

The corpus has access limitations (Table 1, Section 4.2). Saudi Arabia's draft Responsible AI Policy and National Strategy for Data and AI were retrieved only via secondary sources. China's NMPA notice was accessed only as a cover notice (the technical guideline was not obtained), and NHC's 84-scenario guidance was read in Chinese without professional translation. None of China's ten scored cells reached high confidence; a sensitivity band on the five low-confidence cells (Section 4.2) places China's raw score between 5 and 11 of 20, with China remaining the largest gap across the full band. Saudi Arabia's secondary-sourced cells (Table 1) should still be treated as lower-bound estimates.

The RTGI is novel and not previously externally validated. Equal- and alternate-weighted variants yield identical rank ordering, with Saudi Arabia now trailing the EU rather than tying it under both schemes. The re-executed 1000-draw sensitivity sweep (Section 5.3, Fig. 3) shows China's largest-gap position holding in 99.3% of draws, and the full baseline order reproduced in 53.5% of draws (median Spearman rho = 1.000 against baseline) now that the Saudi Arabia-EU tie is resolved. None of this substitutes for external validation against enforcement activity or incident rates.

The analysis covers nineteen documents across four jurisdictions and two normative anchors, scored on a ten-dimension framework reflecting team choices; a different framework could yield different results. This is a cross-sectional snapshot; Saudi Arabia's policy remained in draft consultation and could change.

## 8 Conclusion

This study developed a ten-dimension governance scoring framework, benchmarked against WHO's 2024 large multi-modal model guidance and the EU AI Act, and applied it to Saudi Arabia, the EU, the United States, and China, with reliability assessed via single-coder self-consistency (a genuine second coder was unavailable for this solo-authored study) and a separate construct-validity review. Saudi Arabia scored 15 of 20, second only to the European Union (16) and ahead of the United States (14) and China (6, sensitivity band 5–11 accounting for translation limitations). The RTGI confirmed this ordering under equal and alternate weighting. Four priority gaps emerged: independent third-party conformity assessment, health data governance and interoperability, codified ethical principles with liability and redress mechanisms, and workforce capacity-building, each present in a comparator's binding instruments but absent or internally scoped in Saudi Arabia's. Saudi Arabia's AI health governance appears further along the move from voluntary principles to binding rules than GCC-focused literature suggests, with these four gaps marking where closing them would substantially reduce the measured governance gap relative to the strongest benchmark within the dimensions assessed. The absence of independent inter-rater verification remains the study's principal limitation.

## Declaration of generative AI use

A generative AI tool (Claude, Anthropic) was used during preparation of this manuscript to assist with document formatting, reference-list consistency checks, and language editing. The author reviewed all AI-assisted output and takes full responsibility for the accuracy and integrity of the content; the study conception, analysis, interpretation, and conclusions are his own.

## CRediT authorship contribution statement

**Ahmed Alqheedan:** Writing – review & editing, Writing – original draft, Visualization, Validation, Supervision, Software, Resources, Project administration, Methodology, Investigation, Funding acquisition, Formal analysis, Data curation, Conceptualization.

## Consent to publish

Not applicable. This manuscript does not contain any individual person's data, images, or videos.

## Ethics approval and consent to participate

Not applicable. This study analyzed publicly available policy and regulatory documents; it did not involve human participants, human data, or animals, and therefore did not require ethics committee approval or participant consent.

## Ethics declaration

The author(s) declare(s) that the study does not involve humans nor animal subjects.

## Funding

This research did not receive any specific grant from funding agencies in the public, commercial, or not-for-profit sectors.

## Declaration of competing interest

The author declares that he has no known competing financial interests or personal relationships that could have appeared to influence the work reported in this paper.

## Data Availability

All primary policy and regulatory documents analyzed in this study are publicly available from their originating regulator or standard-setting body; full citations, including DOIs or URLs where issued by the source, are given in the reference list, and a complete document inventory with retrieval status, format, source URL, and official document date (with cited basis for each date) is provided in Supplementary Table S2. The derived analytical datasets generated during this study, namely the full 40-cell governance scoring matrix with per-cell citations and rationale (Supplementary Table S1), the RTGI computations, the single-coder self-consistency reliability check, and the RTGI weight-sensitivity draws, are included in this published article and its supplementary information files. No restricted-access or third-party-licensed data were used.
